# Factors influencing regular exercise in young women: a survey study assessing the preferences and motivators for aerobic and muscle-strengthening exercise

**DOI:** 10.1080/21642850.2025.2465613

**Published:** 2025-02-18

**Authors:** Chloe S. Jones, Katherine E. Spring, Danielle D. Wadsworth

**Affiliations:** a School of Kinesiology, Auburn University, Auburn, AL, USA; b Pennington Biomedical Research, Baton Rouge, LA, USA

**Keywords:** Exercise motivation, exercise adherence, self-regulation, resistance exercise

## Abstract

Young adult women have unique barriers to exercise, but assessing the psychological and behavioral strategies of women who overcome these barriers may be beneficial for informing future exercise programs. This study assessed differences in motives and self-regulation by type (aerobic or muscle-strengthening) and frequency of exercise, along with preferences for exercise amongst young adult women who exercise regularly.

**Methods:**

A survey assessed exercise frequency (International Physical Activity Questionnaire), intensity (Borg’s scale), type, and social and physical preferences. The Exercise Motivations Inventory-2 measured motivators for exercise and the Physical Activity Self-Regulation Scale measured self-regulation. Characteristics and preferences for exercise were examined using descriptives and frequencies. MANOVAs assessed differences in motivators and self-regulation by frequency of exercise, and regression analyses assessed differences in exercise predictors by type.

**Results:**

The sample consisted of 269 women ages 18–34 (66.5% White), of whom 80.3% met the national guidelines for aerobic exercise, 78.4% met the guidelines for muscle-strengthening exercise, and 32.3% identified resistance exercise as their preferred type of exercise. Weight management and self-regulation (*p* = .021, R^2^ = .073) were the strongest predictors of aerobic exercise. Positive health, strength and endurance, and self-regulation (*p* = .023, R^2^ = .161) were predictors of muscle-strengthening exercise. Women who participated in high amounts of aerobic exercise were motivated by interpersonal factors while psychological factors motivated high frequency of muscle-strengthening exercise.

**Conclusions:**

Programs for young adult women should consider incorporating resistance exercise as this study shows it may evoke motivation that could lead to regular participation. Self-regulation strategies were associated with adherence to both aerobic and muscle-strengthening exercises, highlighting the potential importance for inclusion in future interventions.

## Introduction

Efforts to improve health outcomes in the U.S. through increasing physical activity (PA) have been employed for decades (Physical Activity Guidelines Advisory Committee, 2018; U.S. Department of Health and Human Services, 2018; Physical Activity [Internet], 2022). Despite attempts to increase participation in PA, only 22.7% of American adults met the national guidelines for aerobic exercise, 6.8% for muscle-strengthening exercises, and 24.2% for both in 2020 (Elgaddal et al., 2022). A larger portion of men (28.3%) perform PA than women (20.4%), and this trend is present across the lifespan (Elgaddal et al., 2022; Hickey & Mason, 2017; Nuzzo, 2023; Trost et al., 2002). Lack of time is a consistent barrier to exercise reported in women (Biederman et al., 2021; Kowalczyk et al., 2017; Leone & Ward, 2013). Young adult women in particular face increased obstacles in comparison to middle-aged and older women as they are in a period of many life transitions such as moving away from home, getting married, beginning new careers, and starting a family, which can negatively impact their exercise participation (Brown et al., 2009; Hickey & Mason, 2017).

It is critical to address exercise behaviors during this period as health behaviors adopted in this stage of life can impact long-term health outcomes (Gooding et al., 2020). Additionally, during young adulthood, women experience their largest weight gain (Williamson et al., 1990), increasing the chances of developing comorbidities such as obesity and diabetes (Chen et al., 2019; Zheng et al., 2017). To gain understanding of how to increase exercise habits in young adult women, exercise preferences have been assessed in young adults including college students (Box et al., 2019; Burke et al., 2006; Egli et al., 2011; Kilpatrick et al., 2005; Othman et al., 2022; Reading & LaRose, 2018), and studies show that young women like to primarily participate in aerobic exercises (Othman et al., 2022). Muscle-strengthening exercises such as resistance exercise, were preferred sometimes as an additional exercise to aerobic exercise (Reading & LaRose, 2018). This information shows that this population has interest in both types of exercises, but national statistics show a greater number of women participate in aerobic PA versus muscle-strengthening PA (29.6% vs. 3.5%) (Survey NHI, 2018). Previous research surrounding preferences for exercise in young women has focused on the social and physical context as well as preferences for intensity (Burke et al., 2006; Doyle et al., 2019; Othman et al., 2022). Evidence for social and physical context have been mixed as Burke et al., found that collegiate-aged women preferred unstructured exercise with others (Burke et al., 2006). While Othman et al., concluded that most of the young women in their sample liked to exercise alone with a structured program (Othman et al., 2022), and Doyle and colleagues suggested young women preferred supervised group exercise (Doyle et al., 2019). These studies show that social and physical context can vary by individual (Burke et al., 2006; Doyle et al., 2019; Othman et al., 2022). In regard to intensity, studies have shown that young women preferred low to moderate-intensity exercise (Othman et al., 2022), but this research mainly reflects aerobic-based exercise only. Although this research provides some information about where and with whom young adults exercise, questions remain about what motivates them to participate in aerobic or muscle-strengthening exercise and if this motivation differs between the two types of exercise. Motives for exercise are important to assess as higher amounts of motivation from inherent satisfaction or enjoyment, defined as intrinsic motivation, are associated with exercise adherence (Teixeira et al., 2012). Intrinsic motivation is a component of the Self-Determination Theory (SDT) which proposes that an individual’s self-control over a behavior aligns with their level of motivation to perform the behavior (Deci & Ryan, 1985). Also, frequent practices utilized to sustain exercise behavior, such as self-regulatory behaviors identified in Social Cognitive Theory (SCT), should be explored and integrated into interventions aiming to increase exercise adherence. Self-regulation is defined as using psychological functions to self-direct a behavior (Bandura, 1991), and has also been linked to exercise adherence (Ahn et al., 2016; Gell & Wadsworth, 2014).

Considering the risk of worsening physical health of women during young adulthood as well as their additional obstacles to having an active lifestyle, increased efforts should focus on offering support to young women to establish practical and sustainable exercise habits. Previous studies that assessed exercise preferences in young women mainly focused on the social and physical context of the behavior (Burke et al., 2006; Doyle et al., 2019; Othman et al., 2022). Although some papers assessed motives for exercise (Egli et al., 2011; Kilpatrick et al., 2005), they did not screen for length of exercise participation, and all results may not reflect long-term exercise behaviors. Understanding and identifying motives and behavioral practices that predict regular exercise behavior for aerobic and muscle-strengthening exercise have been underexplored in this population. Also, with the goal of increasing exercise frequency in women, an assessment of motivation amongst those who exercise more or less frequently is warranted. Therefore, this study aims to identify motivators and behavioral practices that predict regular exercise by type and frequency and the preference for type of exercise in young adult women between the ages of 18–34 years who regularly exercise.

## Materials & methods

### Participants

This study sought women between the ages of 18–34 years who were regular exercisers defined as those that exercised at least three days per week for the past three months based on ACSM’s pre-screening guidelines for exercise testing (Liguori, 2020). Women were excluded if they were outside of the age range or did not meet the requirement of a regular exerciser. The sample was a majority of White women (66.5%), who were single (56.1%), without children (74.0%), and highly educated (75.1% with degrees) (See Table 1). Participants averaged 27.04 years (*SD* = 4.69) and nearly 50 percent had a normal weight according to their body mass index.

**Table 1.** Participant demographics.

	Sample size	Mean ± SD or Frequency (%)
Age (years)	269	27.04 ± 4.69
Height (m)	269	1.65 ± 0.09
Weight (kg)	263	68.83 ± 15.22
BMI (kg/m^2^)	263	25.22 ± 5.14
Race White or Caucasian Black or African American Asian Hispanic/Latina Native American/Alaskan Native Biracial Native Hawaiian/ Pacific Islander	269	179 (66.5) 48 (17.8) 19 (7.1) 16 (5.9) 3 (1.1) 3 (1.1) 1 (0.4)
Marital Status Single Married Divorced/Separated Engaged Cohabitating	269	151 (56.1) 77 (28.6) 5 (1.9) 10 (3.7) 26 (9.7)
Children/Dependents 0 1 2 ≥3	269	199 (74.0) 34 (12.6) 22 (8.2) 14 (5.2)
Education High School Degree Some College Associate’s Degree Bachelor’s Degree Graduate/Professional Degree	269	20 (7.4) 47 (17.5) 10 (3.7) 96 (35.7) 96 (35.7)
Income <$29,999 $30,000-49,999 $50,000-74,999 $75,000-99,999 ≥$100,000	268	78 (29.1) 37 (13.8) 52 (19.4) 49 (18.3) 52 (19.5)
Employment Full-time Part-time Student Unemployed	269	159 (59.1) 20 (7.4) 76 (28.3) 14 (5.2)

Notes: Body mass index = BMI.

### Study design and procedures

This study was a cross-sectional research design and data were collected via distribution of an online survey (Qualtrics, Provo, UT). The access link to the survey was available from November 2021-June 2022. Convenience sampling was used as physical and digital flyers were distributed at local fitness/recreation facilities and on social media (e.g. Facebook, Instagram, and LinkedIn). Additionally, targeted emails and messages were sent to prospective participants and colleagues within the primary investigator’s (PI) professional and social network. Interested participants accessed the link and were instructed to read and agreed or disagreed to the informed consent. If participants agreed to the study, eligibility was confirmed on the next screen via two questions confirming the age and status of exercise. If eligible, participants continued to the next screen to complete the entirety of the survey. The survey consisted of a mix of multiple choice, select all, Likert scales, and one open-ended question detailed below. All data were collected anonymously and survey completion lasted an average of 25.97 minutes. No compensation was provided for participants. All study procedures were approved by the Auburn University Institutional Review Board.

### Measures

Women completed the survey beginning with demographic questions about race/ethnicity, age, height, weight, marital status, children/dependents, employment status, education, and gross income. Ten questions asked about their preferred exercise intensity (Borg Scale 1-10), type (all modes of exercise, preferred mode of exercise), time (duration and time of day), social preferences (e.g. alone, group-based, etc.), and primary location of exercise. All preference questions were multiple choice except for all modes of exercise participants complete, in which they selected all that applied. The International Physical Activity Questionnaire-long (IPAQ-long) measured the amount of aerobic and muscle-strengthening physical activity. One open-ended question was included inquiring why the participants preferred the primary mode of exercise they selected. Other measures and questionnaires are detailed below.

#### Exercise participation

Aerobic and muscle-strengthening activities were quantified using the IPAQ-long. This questionnaire is a valid and reliable tool for measuring PA in adults aged 18–65 years (r = 0.30, r = .80, respectively) (Craig et al., 2003). Only section four of the questionnaire was used, pertaining to aerobic leisure-time PA over the past seven days, due to this form of PA aligning most closely with exercise. Aerobic leisure-time PA was summed into weekly MET-minutes, and categories were defined as low (< 600 MET-minutes), moderate (600-2999 MET-minutes), and high volume (≥ 3000 MET-minutes) (Forde, 2018). The following question was added and resembled the original version of the IPAQ-long (Craig et al., 2003): ‘During the last seven days, on how many days did you perform muscle-strengthening activities involving all major muscle groups at a moderate-intensity or greater?’ Muscle-strengthening leisure-time PA was summed into days/week over the past seven days. Physical activity categories were defined as low (0-1 d/week), moderate (2-4 days/week), and high frequency (5-7 days/week). Although this addition of questions has not been validated, it has been used as a measure of muscle-strengthening in past studies (Jones et al., 2023; Shannon et al., 2023).

#### Motivation for exercise

The Exercise Motivations Inventory-2 (EMI-2) questionnaire was used to assess psychological, interpersonal, health-related, body-related, and fitness-related motives for exercise (Ingledew et al., 2009; Markland & Ingledew, 1997). This questionnaire comprises 51 statements divided into 14 subscales, shown in Table 1. Participants responded to the statements about reasons they exercised on a scale of 0-5, with a score of 0 indicating the statement is ‘not true at all’ to 5 indicating the statement is ‘very true’. Mean scores for each of the 14 subscales were averaged, with a higher score indicating more motivation to perform exercise. Previous studies confirm EMI-2 as valid and reliable (Markland & Ingledew, 1997; Quindry et al., 2011). Cronbach’s alpha for the current study showed acceptable internal consistency (α = .89) (Table 2).

**Table 2.** EMI-2 categories listed by thematic subscales.

Thematic Category (EMI-2 Subscale)	No. of Items (51 total)	Sample Questionnaire Item
Psychological Motives		
Enjoyment	4	Because I enjoy the feeling of exerting myself
Challenge	4	To give me goals to work to
Revitalization	3	Because it makes me feel good
Stress Management	4	Because it helps reduce tension
Interpersonal Motives		
Affiliation	4	To spend time with friends
Competition	4	Because I like trying to win in physical activities
Social Recognition	4	To show my worth to others
Health Related Motives		
Health Pressures	3	Because my doctor advised me to exercise
Ill-Health Avoidance	3	To prevent health problems
Positive Health	3	To have a healthy body
Body Related Motives		
Appearance	4	To look more attractive
Weight Management	4	To stay slim
Fitness Motives		
Nimbleness	3	To stay/become more agile
Strength/Endurance	4	To increase my endurance

Notes: Exercise Motivations Inventory = EMI.

#### Self-regulation of exercise

The Physical Activity Self-Regulation Scale-12 item (PASR-12) measured the use of self-regulatory practices such as goal-setting, self-monitoring, time-management, eliciting social support, reinforcements, and relapse prevention to sustain exercise (Umstattd et al., 2009). Participants scored the frequency of each using a 5-point Likert scale ranging from ‘never’ to ‘very often.’ Scores from all 12 items were summed and ranged from 12-60, with higher scores demonstrating increased use of self-regulation strategies to maintain exercise. Structural and construct validities were previously established using confirmatory factor analysis (Umstattd et al., 2009), and internal consistency was deemed acceptable based on the reliability coefficient in the current study (α = .82).

### Data analysis

Survey data were analyzed using SPSS Statistics for Windows, version 28.0 (IBM; Armonk, NY). An a priori sample size indicated a range of 84–199 participants depending on the statistical test used to achieve .80 power with an alpha level of .05 and an effect size range of .15-.25. Means and standard deviations were calculated for continuous variables and frequencies were used to represent categorical variables. MANOVAs assessed differences in motives and self-regulation based on frequency of aerobic and muscle-strengthening exercise individually. Bonferroni post-hoc analyses assessed differences between the three groups (low, moderate, and high) for each exercise type. Forward stepwise linear regressions examined whether motivators and self-regulation predicted the amount of exercise performed for aerobic and muscle-strengthening exercises. All 14 subscales of the EMI-2 and the PASR-12 scores were entered into separate models for aerobic and muscle-strengthening. Alpha level was set at .05 a priori for all tests. All statistical assumptions were met for the aerobic exercise MANOVA. For the muscle-strengthening MANOVA, sphericity was violated as the Box’s Test of Equality was statistically significant (*p* = .003), therefore a Pillai’s Trace correction was used. Homogeneity was met for the follow-up statistics based on Levene’s Test of Equality.

Inductive coding was used to analyze the open-ended question. This approach was selected to allow the investigators to derive codes based on the data without any preconceived notions about the outcomes. This was an iterative process involving the PI and a secondary investigator (KES). Initially, each investigator performed a thematic analysis individually by reading the responses and applying a first round of coding using words or phrases that described the concept of the data (Saldaña, 2015). Next, across two meetings, the two investigators reviewed and discussed their interpretations and discrepancies of their codes renaming some codes to better reflect the concepts from the participants (Saldaña, 2015). After reaching a consensus on final codes, investigators individually applied the refined codes to the remainder of the data responses. At the last meeting, the two investigators collaboratively reviewed the final codes to ensure there were no discrepancies and calculated the most frequently reported code for each type of exercise: aerobic and muscle-strengthening.

## Results

### Exercise participation and preferences

In total, 463 women responded to the survey. After screening, 194 participants were removed due to the following reasons: did not consent (n = 4), not a regular exerciser (n = 16), not within the age range (n = 7), or initiated but did not complete the entire survey (n = 167). The final sample size contained 269 women. The physical and contextual characteristics of the participants are provided in Table 3. IPAQ-long scores showed nearly 80% of the women met the national PA guidelines for aerobic PA (≥ 600 MET-minutes/week), about 78% met the national PA guidelines for muscle-strengthening activities (full body muscle-strengthening activities ≥ 2 d/week), and 61% of the women met the guidelines for both. About half of the sample (56.3%) allotted 30–60 minutes to complete their daily exercise, which was most likely to be completed alone (70.3%), in a gym facility (50.4%), at a moderate-intensity (*M* = 6.94, *SD* = 1.42), and in the evening hours (52.7%).

**Table 3.** Exercise characteristics and context.

	Sample Size	Mean ± SD or Frequency (%)
Average Aerobic PA (MET-mins) Low (<600) Moderate (600-2999) High (≥3000)	269	2339.96 ± 1867.61 53 (19.7) 131 (48.7) 85 (31.6)
Average MS PA (days/week) Low (0-1) Moderate (2-4) High (5-7)	269	3.09 ± 1.88 58 (21.6) 149 (55.4) 62 (23.0)
Session Duration (minutes) <30 30–60 61–90 91–120 >120	268	13 (4.9) 151 (56.3) 73 (27.2) 26 (9.7) 5 (1.9)
Time of Day Early Morning Morning Afternoon Evening	268	113 (41.4) 86 (31.5) 96 (35.2) 144 (52.7)
Average Intensity (1-10)	268	6.94 ± 1.42
Location Home/Apartment Gym Outdoors Gym Facility Community Center	269	77 (28.7) 55 (20.5) 135 (50.4) 1 (0.4)
Social Context Alone (self-led) Alone (guided) Personal Trainer (in-person) With Partner Group Mixed guided (online and in-person) With Pet	269	136 (50.6) 53 (19.7) 14 (5.2) 23 (8.6) 41 (15.2) 1 (0.4) 1 (0.4)

Notes: physical activity = PA.

**Figure 1.**
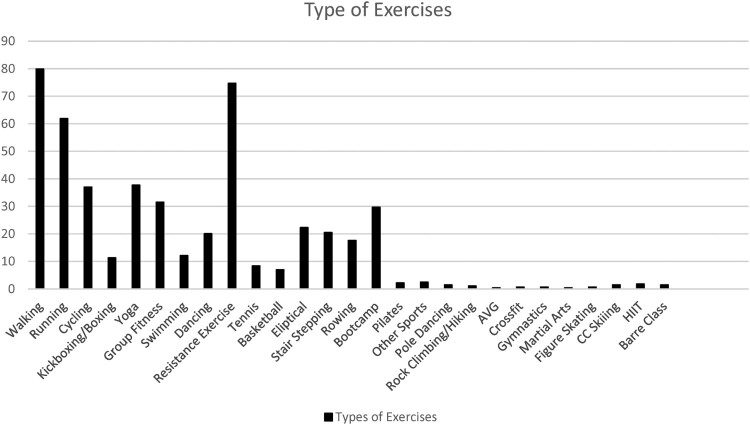
Various modes of exercises performed.

The type of exercises the women performed regularly are illustrated in Figure 1. Walking (79.9%), resistance exercise (74.7%), and running (61.9%) were the most frequently reported modes of exercise. Resistance exercise was the most preferred mode of exercise, indicated by 32.3%, followed by running (20.1%), walking (13.4%), bootcamp style (9.7%), cycling (7.1%), and group fitness (6.3%). All other preferred modes were reported by less than 5% of the sample.

### Qualitative responses of the top motivators for preferred exercise by type

Convenience was the most frequently reported reason to perform aerobic exercises due to ease and familiarity of the movement, no required equipment, and the ability to do it practically anywhere: ‘easiest and most accessible,’ ‘easy to do in my apartment safely (on YouTube),’ and ‘I can do this anytime without equipment.’ Muscle-strengthening exercises were primarily motivated by the desire to increase their physical fitness including building strength and muscle mass. Women stated the following: ‘improving my muscular strength is the goal,’ ‘it makes me feel strong,’ and ‘I build strength and muscle and burn fat at the same time.’

### Differences in motivation and self-regulation by type of exercise

MANOVA results for aerobic exercise are shown in Table 4. We found significant differences between outcomes for the three exercise volume categories (*Λ* = .813, *F* [30, 468] = 1.706, *p *= .012, ηp^2 ^= .099), with challenge being a higher motivator for aerobic exercise in those that performed low ([95% CI, .05–1.09]; *p* = .027, *d *= .45) and high volumes ([95% CI, .03 to .92]; *p* = .032, *d* = .37) versus those who participated in a moderate level. Social recognition ([95% CI, .16–1.12]; *p* = .004, *d *= .47), affiliation ([95% CI, 1.5–1.20]; *p* = .006, *d *= .45), competition ([95% CI, .23–1.37]; *p* = .002, *d* = .48), and self-regulation ([95% CI, 1.80–7.16]; *p* < .001, *d *= .58) were all significantly higher motivators in those who performed high versus moderate volumes. Those who performed high volumes of aerobic exercise (≥ 3000 MET-minutes/week) were significantly more motivated by weight management than those who performed moderate ([95% CI, .04 to .96]; *p* = .028, *d *= .40,) and low ([95% CI, .14–1.30], *p* = .009, *d *= .55) volumes. No other significant differences by volume were found.

**Table 4.** MANOVA – differences in motivators and self-regulation by volume of aerobic exercise.

	Low^a^ (*n* = 49)		Moderate^b^ (*n* = 121)		High^c^ (*n* = 81)				
	M	SD	M	SD	M	SD	F	sig	ηp^2^
Stress Management	3.89	1.27	3.75	1.18	3.91	1.08	0.549	.578	.004
Revitalization	3.91	1.20	3.81	1.10	4.06	0.94	1.238	.292	.010
Enjoyment	4.04	1.29	3.80	1.18	3.95	1.19	1.939	.146	.015
Challenge	3.57^b^	1.22	2.99	1.32	3.47^b^	1.25	5.066	.**007**	.039
Social Recognition	1.68	1.53	1.57	1.28	2.21^b^	1.43	5.418	.**005**	.042
Affiliation	2.15	1.68	1.60	1.36	2.28^b^	1.61	5.496	.**005**	.042
Competition	2.11	1.66	1.83	1.59	2.63^b^	1.71	5.782	.**004**	.045
Health Pressures	1.58	1.50	1.44	1.12	1.77	1.34	1.657	.193	.013
Ill-Health avoidance	3.98	1.16	3.80	1.03	3.93	0.99	0.672	.512	.005
Positive Health	4.50	0.73	4.52	0.64	4.53	0.74	0.033	.967	.000
Weight Management	3.05	1.48	3.27	1.40	3.77^a,b^	1.09	5.435	.**005**	.042
Appearance	3.31	1.20	3.14	1.22	3.54	1.15	2.777	.064	.022
Strength & Endurance	4.28	0.89	4.11	0.76	4.23	0.91	0.933	.395	.007
Nimbleness	3.31	1.63	3.11	1.25	3.45	1.26	1.612	.202	.013
									
PASR-12	45.16	8.01	42.48	7.52	46.96^b^	7.91	8.387	**<**.**001**	.063

Notes: ^a^significant difference from low volume; ^b^significant difference from moderate volume. Low = <600 MET-minutes/week; moderate = 600-2999 MET-minutes/week; high = ≥3000 MET-minutes/week.

Table 5 shows MANOVA results for muscle-strengthening exercise across the three frequency groups (low, moderate, or high). The MANOVA showed significant differences (*Ѵ* = .259, *F* [30, 470] = 2.333, *p *< .001, ηp^2 ^= .133) by frequency of training. Follow-up analyses showed high frequency muscle-strengthening exercisers had significantly more motivation from revitalization ([95% CI, .23–1.19]; *p* = .001, *d* = .71), enjoyment ([95% CI, .29–1.35]; *p* < .001, *d* = .73), challenge ([95% CI, .39–1.54]; *p* < .001, *d* = .77), social recognition ([95% CI, .28–1.54]; *p* = .002, *d* = .68), competition ([95% CI, .05–1.56]; *p* = .031, *d* = .48), appearance ([95% CI, .12–1.20]; *p* = .010, *d* = .52), strength and endurance ([95% CI, .45–1.17]; *p* < .001, *d* = .92), and nimbleness ([95% CI, .26–1.45]; *p* = .002, *d* = .65), than low-frequency exercisers. Women who performed muscle-strengthening exercises at a moderate versus a low-frequency had significantly higher motives from challenge ([95% CI, .08–1.05]; *p* = .016, *d *= .44) appearance ([95% CI, .02 to .94]; *p* = .035, *d* = .39), strength and endurance ([95% CI, .30 to .91]; *p* < .001, *d* = .69), and nimbleness ([95% CI, .15–1.16]; *p* = .006, *d* = .50). Self-regulation significantly increased as the frequency of muscle-strengthening exercise increased low to moderate: ([95% CI, – 6.79 to – .98]; *p* = .004, *d* = .49); low to high: ([95% CI, – 11.16 to – 4.30]; *p* < .001 *d *= 1.00); moderate to high: ([95% CI, – 6.70 to – .99]; *p* = .004, *d* = .54). No other significant differences were detected.

**Table 5.** MANOVA – differences in motivators and self-regulation by frequency of muscle-strengthening exercise.

	Low^a^ (*n* = 55)		Moderate^b^ (*n* = 138)		High^c^ (*n* = 58)				
	M	SD	M	SD	M	SD	F	sig	ηp^2^
Stress Management	3.67	1.21	3.76	1.20	4.16	0.99	3.114	.**046**	.024
Revitalization	3.56	1.17	3.90	1.09	4.27^a^	0.79	6.511	.**002**	.050
Enjoyment	3.53	1.23	3.95	1.20	4.34^a^	1.00	6.950	.**001**	.053
Challenge	2.72	1.31	3.29^a^	1.27	3.69^a^	1.20	8.296	**<**.**001**	.063
Social Recognition	1.36	1.32	1.78	1.40	2.27^a^	1.37	6.246	.**002**	.048
Affiliation	1.73	1.56	1.89	1.51	2.19	1.58	1.329	.266	.011
Competition	1.81	1.67	2.08	1.63	2.62^a^	1.71	3.570	.**030**	.028
Health Pressures	1.44	1.30	1.65	1.28	1.52	1.26	0.584	.558	.005
Ill-Health avoidance	3.80	1.01	3.85	1.08	4.03	0.97	0.816	.444	.007
Positive Health	4.49	0.67	4.48	0.72	4.63	0.64	1.014	.364	.008
Weight Management	3.26	1.42	3.40	1.32	3.49	1.36	0.412	.663	.003
Appearance	2.89	1.35	3.37^a^	1.11	3.55^a^	1.18	4.809	.**009**	.037
Strength & Endurance	3.66	1.01	4.27^a^	0.71	4.47^a^	0.71	16.770	**<**.**001**	.119
Nimbleness	2.70	1.30	3.35^a^	1.30	3.55^a^	1.32	6.931	.**001**	.053
PASR-12	40.53	8.43	44.41^a^	7.50	48.26^a,b^	6.83	14.743	**<**.**001**	.106

Notes: ^a^significant difference from low volume; ^b^significant difference from moderate volume. Low = 0-1 d/week; moderate = 2-4 days/week; high = 5-7 days/week.

### Contributors to aerobic and muscle-strengthening exercises

A forward stepwise linear regression assessed whether motivational factors or self-regulation predicted aerobic exercise volume or muscle-strengthening frequency (Table 6). Volume of aerobic exercise was significantly influenced by self-regulation (*B* = 53.743, β = .229, *t* = 3.716, *p* < .001) and self-regulation plus weight management (*B* = 200.290, β = .145, *t* = 2.315, *p* = .021). At step 1 and step 2 of the analysis, the overall regression equation was significant for self-regulation (*F*_1, 249_ = 13.810, *p* < .001) accounting for 5.3% of the variance (R^2^ = .053, Adjusted R^2^ = .049) and self-regulation and weight management (*F*_2, 248_ = 9.706, *p* < .001) explaining 7.3% of the variance (R^2^ = .073, Adjusted R^2^ = .065).

**Table 6.** Regression for contributors to aerobic and muscle-strengthening exercises.

Model		B	R Square	R Square Change	95% CI	Sig.
**Aerobic**						
1	(Constant)	−21.338	.053	.053	(−1307.534, 1264.859)	**<**.**001**
	PASR-12	53.743			(25.260, 82.226)	
2	(Constant)	−393.145	.073	.020	(−1706.903, 920.613)	.**021**
	PASR-12	46.832			(17.989, 75.675)	
	Weight Management	200.290		0.145	(29.889, 370.691)	
**Muscle strengthening**						
1	(Constant)	-.364	.106	.106	(−1.632, .904)	**<**.**001**
	PASR-12	.077		.037	(.049, .105)	
2	(Constant)	−1.377	.143	.018	(−2.761, .007)	.**001**
	PASR-12	.054			(.023, .085)	
	Strength & Endurance	.494			(.198, .789)	
3	(Constant)	-.337	.161		(−1.974, 1.300)	.**023**
	PASR-12	.060			(.029, .091)	
	Strength & Endurance	.638			(.319, .956)	
	Positive Health	-.428			(-.796, – .060)	

Notes: The level of significance is *p* < 0.05. Significant *p*-values are bolded. B = unstandardized betas.

Excluded variables for aerobic: stress management, revitalization, enjoyment, challenge, social recognition, affiliation, competition, health pressures, ill-health avoidance, positive health, appearance, strength & endurance, nimbleness.

Excluded variables for muscle-strengthening: stress management, revitalization, enjoyment, challenge, social recognition, affiliation, competition, health pressures, ill-health avoidance, weight management, appearance, nimbleness.

For muscle-strengthening exercise, the amount of exercise was significantly influenced by self-regulation (*B* = .077, β = .325, *t* = 5.419, *p* < .001), strength and endurance (*B* = .494, β = .217, *t* = 3.286, *p* = .001), and positive health (*B* = -.428, β = -.156, *t* = −2.293, *p* = .023). At steps 1, 2, and 3 of the analysis, the overall regression equation was significant for self-regulation (*F*_1,249_ = 29.370, *p* < .001), self-regulation, and strength and endurance (*F*_2, 248_ = 20.661, *p* < .001), and self-regulation, strength and endurance, and positive health (*F*_3, 247_ = 15.763, *p* < .001). Self-regulation explained 10.6% of the variance (R^2^ = .106, Adjusted R^2^ = .102), self-regulation, and strength and endurance explained 14.3% of the variance (R^2^ = .143, Adjusted R^2^ = .136), and self-regulation, strength and endurance, and positive health explained 16.1% (R^2^ = .161, Adjusted R^2^ = .150).

## Discussion

The purpose of this study was to explore preferences, motives, and behavioral practices for aerobic and muscle-strengthening exercise and frequency of exercise in young women who exercise regularly. Several findings emerged regarding social context for exercise, preference for resistance exercise, differences in motivation by frequency of exercise, differences in predictors for aerobic and muscle-strengthening exercise, and the use of self-regulation strategies for both types of exercise.

Most women in this study preferred to exercise alone and unsupervised. Although this finding overlaps with results from Othman et al. (2022), it contradicts previous findings that women tend to feel uncomfortable exercising alone (Nuzzo, 2023), and prefer group settings for aerobic and weight training activities (Burke et al., 2006). Women have stated that exercising in group settings help hold them accountable, maintain commitment, and is a social opportunity with other women (Carter & Alexander, 2020; Kinsey et al., 2019; Vasudevan & Ford, 2022). However, it is possible that these women’s exercise regimens in this study are competing with other life events and obligations prompting them to exercise when it is most convenient. Women in the current study selected multiple times of day they exercise, ranging from early morning to the evening, highlighting the importance of schedule flexibility in maintaining their exercise regimens long-term. Programming future exercise interventions for this age group should consider the possibility of variability in their daily and weekly schedules and plan for flexibility.

Nearly 32% of the participants identified resistance exercise as their preferred mode of exercise over aerobic exercises such as walking, running, and cycling. Jones et al. found similar findings from interviews with young active women (Jones et al., 2023), but overall, our results contradict a large portion of literature suggesting women prefer aerobic activities (i.e. walking, dance, swimming) or yoga over muscle-strengthening activities (Abrantes et al., 2011; Busch et al., 2016; Doyle et al., 2019), and use cardiovascular equipment more than strength equipment in gym settings (Rapport et al., 2018; Salvatore & Marecek, 2010). Preference for aerobic exercises has been linked to hesitation in performing resistance exercises in unsupervised settings (Nuzzo, 2023; Rodriguez-Hernandez et al., 2022). A recent systematic review of motivators and barriers for strength training in women highlighted that historically, women have not been efficiently taught how to perform strength training exercises and receive minimal supervision and encouragement to perform them (Vasudevan & Ford, 2022). Further reluctance has stemmed from gender-based social stigmas (e.g. being a ‘man’s’ exercise, looking manly or bulky), the dominance of strength equipment and areas by men along with feeling unwelcomed, unwanted attention, and verbal discouragement when in those areas (Vasudevan & Ford, 2022). Our results show a potential shift in this ideology and support previous findings demonstrating an increase in women participating in muscle-strengthening activities (Jones et al., 2023; Kekäläinen et al., 2018; Kinsey et al., 2019; Winett et al., 2015). However, this study is one of the first cross-sectional studies to distinctly identify resistance exercise as the preferred mode. This shift may be due to more frequent observance of women performing resistance exercises on social media, friends, and other women in gym settings (Jones et al., 2023).

Predictors for aerobic and muscle-strengthening exercises differed in this sample except for self-regulation. Our findings indicate weight management is a large predictor for aerobic exercise. Preserving one’s health and improving physical fitness were the primary predictors for muscle-strengthening exercise, which was also supported by the open-ended responses (e.g. building strength and muscle mass). These findings are similar to other results in the literature for women (Benau et al., 2019; Ednie & Stibor, 2017; Jones et al., 2023; Nuzzo, 2023). Markland and Ingledew (2007) suggest that external regulators such as weight management and appearance have a moderate effect on exercise participation and show little contribution to long-term maintenance for exercise (Ingledew et al., 2009; Markland & Ingledew, 2007). However, our results demonstrate that weight management and self-regulation strategies enable young women to meet aerobic exercise recommendations. Other factors, such as the convenience, ease, and lack of equipment needed to perform aerobic activities expressed in the short responses by the participants, may also contribute to aerobic exercise adherence. Health and fitness-related motives were the strongest predictors for muscle-strengthening exercise, and these motives have been associated with identified regulation (Teixeira et al., 2012). Identified regulation proposes that someone performs a regular behavior (i.e. exercise) because it aligns with their personal goals and values (Ingledew & Markland, 2008; Ryan & Deci, 2017). Although intrinsic motivation has the greatest association with exercise adherence (Teixeira et al., 2012), the results of the current study suggest the combination of muscle-strengthening exercise and heightened self-regulation allow these women to more frequently align their health principles with their actual behavior resulting in sustained exercise.

Our results also reveal that motivation might depend on exercise frequency and type. Women who participated in high volumes of aerobic exercise were more motivated by interpersonal factors (e.g. social recognition, affiliation, and competition) than those who participated in low volumes of aerobic exercise. Additionally, challenge (psychological motive) and weight management (body-related motive) were significantly higher motivators for those that exceeded PA recommendations than those that did not. Again, these results are unsurprising as evidence exists stating women like to exercise in group settings and are driven by their body appearance (Egli et al., 2011; Kilpatrick et al., 2005; Othman et al., 2022).

High frequency of muscle-strengthening exercise was largely motivated by psychological (enjoyment, challenge, and revitalization), interpersonal (social recognition and competition), fitness (strength and endurance and nimbleness), and body-related motives (appearance). Muscle-strengthening exercise has been linked to enjoyment, which is intrinsically motivated, but in samples with unknown or short-term lengths of adherence to exercise (Hornbuckle et al., 2021; Rhodes et al., 2017; Rodriguez-Hernandez et al., 2022). Box and colleagues reported resistance training exercisers were more intrinsically motivated than group or aerobic exercisers (Box et al., 2019). Our results expand this evidence by demonstrating the connection between muscle-strengthening exercise, intrinsic motivation, and regular exercisers. Open-ended responses revealed that the women preferred muscle-strengthening exercise to increase their strength, muscle mass, and it makes them ‘*feel strong*,’ which supports the significant fitness motives identified. Future research should focus on methods to increase intrinsic motivation for muscle-strengthening exercises, as this may help facilitate more regular exercise.

Finally, our results indicate that self-regulation was a predictor of sustained exercise for aerobic and muscle-strengthening exercises. This result is unsurprising as strategies such as planning and goal-setting (Ahn et al., 2016; Huberty et al., 2008; Kinsey et al., 2019; Winett et al., 2015), time-management (Gell & Wadsworth, 2014), eliciting social support or accessing resources (Kinsey et al., 2019; Kirchhoff et al., 2008; Winett et al., 2015), and coping planning (relapse prevention) (Gerber et al., 2015; Stadler et al., 2009) have been linked to exercise adherence before. Of these studies, only three were randomized controlled trials (Huberty et al., 2008; Stadler et al., 2009; Winett et al., 2015). Results from Huberty et al., showed that those who adhered to their exercise regimen (maintenance for ≥ 1 year) stated that planning, goal-setting, social support, and accessing other resources for knowledge contributed to their success (Huberty et al., 2008). Participants from the Winett study reported use of self-regulatory strategies led to 77.5% adherence to resistance exercise (twice per week) after 6 months of unsupervised training and 53.1% after 12 months of unsupervised training (Winett et al., 2015). These studies highlight the need and successful utility of teaching and implementing self-regulation. Therefore, self-regulation strategies in exercise interventions in young adult women should be explored further as it is a shared commonality in predicting and promoting aerobic and muscle-strengthening exercise.

### Strengths and limitations

This study has several notable strengths. First, this study identified resistance exercise as the preferred mode of exercise, and regular participation was driven by psychological motives, fitness-related motives, and self-regulation. Self-regulation strategies should be implemented more frequently in future exercise interventions as research and the current study show its positive utility in both types of exercise and its potential to increase chances of adherence despite source of motivation. Secondly, to our knowledge, this is one of the first studies to assess differences in motivation by type of exercise and predictors by volume of exercise in young women. Greater amounts of aerobic exercise were motivated by social aspects, and muscle-strengthening was driven by psychological benefits. This information is pertinent when discussing strategies to transition low-frequency exercisers to moderate – or high-frequency exercisers. More specifically, researchers may benefit from including social connections for aerobic exercise and enjoyable and challenging routines to increase muscle-strengthening exercises maintenance.

Although this study has several strengths, some limitations should be addressed. First, this study used a cross-sectional design which is unable to establish causal effects and assess behaviors over time, and may subject the results to information bias (Wang & Cheng, 2020). Physical activity data was self-reported, which introduces the chances of over or under-estimating PA. The addition of the muscle-strengthening questions on the IPAQ-long has not been validated and therefore cannot confirm that the data was assessed adequately and reliably. However, the shortcomings of surveillance methods for muscle-strengthening exercise have been recognized and the addition of these questions to the IPAQ or Global Physical Activity Questionnaire has been suggested (Shakespear-Druery et al., 2021). Most of this sample were single and without children, potentially increasing the amount of autonomy in their schedules. This decreases the ability to generalize results to women who are married and/or with children. Lastly, nearly two-thirds of the participants were White women, potentially making the results less generalizable to women from underrepresented racial groups. Future surveys should strive to collect data from more racially diverse backgrounds to assess if differences in preferences and motives for both types of exercise exist. These findings are pertinent to programming and tailoring exercise interventions for diverse groups of women.

## Conclusion

This study identified several key components of women who regularly participate in aerobic and muscle-strengthening exercise. With the increased popularity of resistance exercise in women, more exercise studies should consider incorporating muscle-strengthening exercises in interventions. More research is needed to assess the acceptability and its effect on long-term maintenance in women who perform these exercises and what contributes to exercise maintenance. Acknowledging the differences in motivators for aerobic versus muscle-strengthening exercise may help understand participants’ goals and help tailor their exercise regimens to evoke more intrinsic motivation. Lastly, self-regulation persevered as a common facilitator and predictor of regular exercise. Incorporating discussions surrounding the components and use of self-regulation to sustain exercise may lead to increased positive outcomes for long-term exercise.

## Ethics approval statement

This study was approved by the Auburn University Institutional Review Board/Ethics Committee (approval no. 21–450 EX 2110)

## Author contributions

CSJ is the principal investigator for the current study and served the roles in conceptualization, data curation, formal analysis, investigation, methodology, project administration, writing – original draft and review & editing. KES assisted in the roles of formal analysis and writing – review & editing. DDW provided mentorship and served the roles of conceptualization, project administration, and writing – review & editing.

## Informed statement

All participants were provided written informed consent prior to enrollment in the study.

## Institutional review board statement

The study was conducted in accordance with the Declaration of Helsinki and was approved by an Institutional Review Board/Ethics committee. See details under Methods.

The study received an exemption from the Institutional Review Board/Ethics committee. See details under Methods.

## References

[CIT0001] Abrantes, A. M., Battle, C. L., Strong, D. R., Ing, E., Dubreuil, M. E., Gordon, A., & Brown, R. A. (2011). Exercise preferences of patients in substance abuse treatment. *Mental Health and Physical Activity*, *4*(2), 79–87. 10.1016/j.mhpa.2011.08.002 22125581 PMC3224086

[CIT0002] Ahn, J., Jeon, H., & Kwon, S. (2016). Associations between self-regulation, exercise participation, and adherence intention among Korean university students. *Perceptual and Motor Skills*, *123*(1), 324–340. 10.1177/0031512516659874 27450864

[CIT0003] Bandura, A. (1991). Social cognitive theory of self-regulation. *Organizational Behavioral Human Decision Process*, *50*(2), 248–287. 10.1016/0749-5978(91)90022-L

[CIT0004] Benau, E. M., Plumhoff, J., & Timko, C. A. (2019). Women's dieting goals (weight loss, weight maintenance, or not dieting) predict exercise motivation, goals, and engagement in undergraduate women: A self-determination theory framework. *International Journal of Sport and Exercise Psychology*, *17*(6), 553–567. 10.1080/1612197X.2017.1421683

[CIT0005] Biederman, D. J., Sabol, V. K., Thompson, J., Duncan, Q., & Pereira, K. C. (2021). Increasing physical activity with African–American women using Facebook™ and Pedometers. *Public Health Nursing*, *38*(4), 671–674. 10.1111/phn.12876 33682156

[CIT0006] Box, A. G., Feito, Y., Brown, C., & Petruzzello, S. J. (2019). Individual differences influence exercise behavior: How personality, motivation, and behavioral regulation vary among exercise mode preferences. *Heliyon*, *5*(4), e01459. 31065599 10.1016/j.heliyon.2019.e01459 PMC6496506

[CIT0007] Brown, W. J., Heesch, K. C., & Miller, Y. D. (2009). Life events and changing physical activity patterns in women at different life stages. *Annals of Behavioral Medicine*, *37*(3), 294–305. 10.1007/s12160-009-9099-2 19506989

[CIT0008] Burke, S. M., Carron, A. V., & Eys, M. A. (2006). Physical activity context: Preferences of university students. *Psychology of Sport and Exercise*, *7*(1), 1–13. 10.1016/j.psychsport.2005.03.002

[CIT0009] Busch, A. M., Ciccolo, J. T., Puspitasari, A. J., Nosrat, S., Whitworth, J. W., & Stults-Kolehmainen, M. (2016). Preferences for exercise as a treatment for depression. *Mental Health and Physical Activity*, *10*, 68–72. 10.1016/j.mhpa.2015.12.004 27453730 PMC4955620

[CIT0010] Carter, A., & Alexander, A. C. (2020). A qualitative exploration of womens’ experiences who belong to a “fitness community”. *American Journal of Health Education*, *51*(1), 22–30. 10.1080/19325037.2019.1687365

[CIT0011] Chen, C., Ye, Y., Zhang, Y., Pan, X.-F., & Pan, A. (2019). Weight change across adulthood in relation to all cause and cause specific mortality: Prospective cohort study. *BMJ*, 367. 10.1136/bmj.l5584 PMC6812615 31619383

[CIT0012] Craig, C. L., Marshall, A. L., Sjöström, M., Bauman, A. E., Booth, M. L., Ainsworth, B. E., Pratt, M., Ekelund, U. L. F., Yngve, A., Sallis, J. F., & Oja, P. (2003). International physical activity questionnaire: 12-country reliability and validity. *Medicine Science & Sports Exercise*, *35*(8), 1381–1395. 10.1249/01.MSS.0000078924.61453.FB 12900694

[CIT0013] Deci, E. L., & Ryan, R. M. (1985). *Intrinsic motivation and self-determination in human behavior*. Plenum.

[CIT0014] Doyle, C., Khan, A., & Burton, N. (2019). Recreational physical activity context and type preferences among male and female Emirati university students. *International Health*, *11*(6), 507–512. 10.1093/inthealth/ihz002 31220266

[CIT0015] Ednie, A., & Stibor, M. (2017). Influence and interpretation of intrinsic and extrinsic exercise motives. *Journal of Human Sport and Exercise*, *12*(2), 414–425. 10.14198/jhse.2017.122.18

[CIT0016] Egli, T., Bland, H. W., Melton, B. F., & Czech, D. R. (2011). Influence of age, sex, and race on college students’ exercise motivation of physical activity. *Journal of American College Health*, *59*(5), 399–406. 10.1080/07448481.2010.513074 21500059

[CIT0017] Elgaddal, N., Kramarow, E. A., & Reuben, C. (2022). Physical activity among adults aged 18 and over: United States, 2020. *NCHS Data Brief*, *443*, 1–8. 36043905

[CIT0018] Forde, C. (2018). *Scoring the international physical activity questionnaire (IPAQ)*. University of Dublin, 3.

[CIT0019] Gell, N. M., & Wadsworth, D. D. (2014). How do they do it: Working women meeting physical activity recommendations. *American Journal of Health Behavior*, *38*(2), 208–217. 10.5993/AJHB.38.2.6 24765681 PMC4043126

[CIT0020] Gerber, M., Lindwall, M., Brand, S., Lang, C., Elliot, C., & Pühse, U. (2015). Longitudinal relationships between perceived stress, exercise self-regulation and exercise involvement among physically active adolescents. *Journal of Sports Science*, *33*(4), 369–380. 10.1080/02640414.2014.946072 25098842

[CIT0021] Gooding, H. C., Gidding, S. S., Moran, A. E., Redmond, N., Allen, N. B., Bacha, F., et al. (2020). Challenges and opportunities for the prevention and treatment of cardiovascular disease among young adults: Report from a National Heart, Lung, and Blood Institute Working Group. *Journal of the American Heart Association*, *9*(19), e016115. 10.1161/JAHA.120.016115 32993438 PMC7792379

[CIT0022] Hickey, M. E., & Mason, S. E. (2017). Age and gender differences in particpation rates, motivators for, and barriers to exercise. *Modern Psychological Studies*, *22*(2), 3.

[CIT0023] Hornbuckle, L. M., Barroso, C. S., Rauer, A., Jones, C. S., & Winters-Stone, K. M. (2021). “It was just for us”: qualitative evaluation of an exercise intervention for African-American couples. *BMC Public Health*, *21*(1), 838. 10.1186/s12889-021-10659-2 33933048 PMC8087875

[CIT0024] Huberty, J. L., Ransdell, L. B., Sidman, C., Flohr, J. A., Shultz, B., Grosshans, O., & Durrant, L. (2008). Explaining long-term exercise adherence in women who complete a structured exercise program. *Research Quarterly Exercise Sport*, *79*(3), 374–384. 10.1080/02701367.2008.10599501 18816949

[CIT0025] Ingledew, D. K., & Markland, D. (2008). The role of motives in exercise participation. *Psychology and Health*, *23*(7), 807–828. 10.1080/08870440701405704 25160882

[CIT0026] Ingledew, D. K., Markland, D., & Ferguson, E. (2009). Three levels of exercise motivation. *Applied Psychology: Health and Well-Being*, *1*(3), 336–355. 10.1111/j.1758-0854.2009.01015.x

[CIT0027] Jones, C. S., Barroso, C. S., Miossi, L. A., Fitzhugh, E., & Hornbuckle, L. M. (2023). Successful physical activity maintainers: Strategies and characteristics of young African American women. *Women Sport Physical Activity Journal*, *32*(1). 10.1123/wspaj.2022-0077

[CIT0028] Kekäläinen, T., Kokko, K., Tammelin, T., Sipilä, S., & Walker, S. (2018). Motivational characteristics and resistance training in older adults: A randomized controlled trial and 1-year follow-up. *Scandinavian Journal of Medicine and Science Sports*, *28*(11), 2416–2426. 10.1111/sms.13236 29878445

[CIT0029] Kilpatrick, M., Hebert, E., & Bartholomew, J. (2005). College students’ motivation for physical activity: Differentiating men's and women's motives for sport participation and exercise. *Journal of American College Health*, *54*(2), 87–94. 10.3200/JACH.54.2.87-94 16255320

[CIT0030] Kinsey, A. W., Segar, M. L., Barr-Anderson, D. J., Whitt-Glover, M. C., & Affuso, O. (2019). Positive outliers among African American women and the factors associated with long-term physical activity maintenance. *Journal of Racial and Ethnic Health Disparities*, *6*(3), 603–617. 10.1007/s40615-018-00559-4 30644068 PMC6500467

[CIT0031] Kirchhoff, A. C., Elliott, L., Schlichting, J. A., & Chin, M. H. (2008). Strategies for physical activity maintenance in African American women. *American Journal of Health Behavior*, *32*(5), 517–524. 10.5993/AJHB.32.5.7 18241136 PMC2659643

[CIT0032] Kowalczyk, A., Nowicka, M., & Sas-Nowosielski, K. (2017). Age-related differences in motives for and barriers to exercise among women exercising in fitness centers. *The New Educational Review*, *49*(3), 30–39. 10.15804/tner.2017.49.3.02

[CIT0033] Leone, L. A., & Ward, D. S. (2013). A mixed methods comparison of perceived benefits and barriers to exercise between obese and nonobese women. *Journal of Physical Activity and Health*, *10*(4), 461–469. 10.1123/jpah.10.4.461 23714626 PMC3904548

[CIT0034] Liguori, G. (2020). *Medicine ACoS. ACSM's guidelines for exercise testing and prescription*. Lippincott Williams & Wilkins.

[CIT0035] Markland, D., & Ingledew, D. K. (1997). The measurement of exercise motives: Factorial validity and invariance across gender of a revised Exercise Motivations Inventory. *British Journal of Health Psychology*, *2*(4), 361–376. 10.1111/j.2044-8287.1997.tb00549.x

[CIT0036] Markland, D., & Ingledew, D. K. (2007). Exercise participation motives: A self-determination theory perspective.

[CIT0037] Nuzzo, J. L. (2023). Narrative review of sex differences in muscle strength, endurance, activation, size, fiber type, and strength training participation rates, preferences, motivations, injuries, and neuromuscular adaptations. *The Journal of Strength & Conditioning Research*, *37*(2), 494–536. 10.1519/JSC.0000000000004329 36696264

[CIT0038] Othman, M. S., Mat Ludin, A. F., Chen, L. L., Hossain, H., Halim, A., Sameeha, I. I., J, M., et al. (2022). Motivations, barriers and exercise preferences among female undergraduates: A need assessment analysis. *PLoS One*, *17*(2), e0264158. 10.1371/journal.pone.0264158 35226684 PMC8884489

[CIT0039] Physical Activity Guidelines Advisory Committee. (2018). Physical Activity Guidelines Advisory Committee Scientific Report. Washington, D.C; 2018.

[CIT0040] Physical Activity [Internet]. (2022). https://www.who.int/news-room/fact-sheets/detail/physical-activity.

[CIT0041] Quindry, J. C., Yount, D., O'bryant, H., & Rudisill, M. E. (2011). Exercise engagement is differentially motivated by age-dependent factors. *American Journal of Health Behavior*, *35*(3), 334–345. 10.5993/AJHB.35.3.7 21683022

[CIT0042] Rapport, F., Hutchings, H., Doel, M. A., Wells, B., Clement, C., Mellalieu, S., Shubin S., Brown D., Seah R., Wright S., & Sparkes A. (2018). How are university gyms used by staff and students? A mixed-method study exploring gym use, motivation, and communication in three UK gyms. *Societies*, *8*(1), 15. 10.3390/soc8010015

[CIT0043] Reading, J. M., & LaRose, J. G. (2018). Exercise preferences among young adults: Do Men and women want different things? *Journal of American College Health*, *70*(5), 1301–1305. 10.1080/07448481.2020.1803878 PMC8404202 32813629

[CIT0044] Rhodes, R. E., Lubans, D. R., Karunamuni, N., Kennedy, S., & Plotnikoff, R. (2017). Factors associated with participation in resistance training: A systematic review. *British Journal of Sports Medicine*, *51*(20), 1466–1472. 10.1136/bjsports-2016-096950 28404558

[CIT0045] Rodriguez-Hernandez, M. G., McDonald, J. R., Pascoe, D. D., & Wadsworth, D. D. (2022). The effect of a sprint interval and resistance training program on body composition, aerobic fitness, and self-regulation in young women. *European Journal of Sport Sciences*, *1*(4), 13–21. 10.24018/ejsport.2022.1.4.26

[CIT0046] Ryan, R. M., & Deci, E. L. (2017). *Self-determination theory: Basic psychological needs in motivation, development, and wellness*. Guilford Publications.

[CIT0047] Saldaña, J. (2015). *The coding manual for qualitative researchers*. Sage.

[CIT0048] Salvatore, J., & Marecek, J. (2010). Gender in the gym: Evaluation concerns as barriers to women’s weight lifting. *Sex Roles*, *63*(7-8), 556–567. 10.1007/s11199-010-9800-8

[CIT0049] Shakespear-Druery, J., De Cocker, K., Biddle, S. J., Gavilán-Carrera, B., Segura-Jiménez, V., & Bennie, J. (2021). Assessment of muscle-strengthening exercise in public health surveillance for adults: A systematic review. *Preventive Medicine*, *148*, 106566. 10.1016/j.ypmed.2021.106566 33878352

[CIT0050] Shannon, S., Shevlin, M., Brick, N., & Breslin, G. (2023). Frequency, intensity and duration of muscle strengthening activity and associations with mental health. *Journal of Affective Disorders*, *325*, 41–47. 10.1016/j.jad.2022.12.063 36587908

[CIT0051] Stadler, G., Oettingen, G., & Gollwitzer, P. M. (2009). Physical activity in women: Effects of a self-regulation intervention. *American Journal of Prevention Medicine*, *36*(1), 29–34. 10.1016/j.amepre.2008.09.021 18977113

[CIT0052] Survey NHI. (2018). Table A-14a. Age-adjusted percent distributions (with standard errors) of participation in leisure-time aerobic and muscle-strengthening activities that meet the 2008 federal physical activity guidelines among adults aged 18 and over, by selected characteristics: United States.

[CIT0053] Teixeira, P. J., Carraça, E. V., Markland, D., Silva, M. N., & Ryan, R. M. (2012). Exercise, physical activity, and self-determination theory: A systematic review. *International Journal of Behavioral Nutrition and Physical Activity*, *9*(1), 78. 10.1186/1479-5868-9-78 22726453 PMC3441783

[CIT0054] Trost, S. G., Pate, R. R., Sallis, J. F., Freedson, P. S., Taylor, W. C., Dowda, M., & Sirard, J. (2002). Age and gender differences in objectively measured physical activity in youth. *Medical Science & Sports Exercise*, *34*(2), 350–355. 10.1097/00005768-200202000-00025 11828247

[CIT0055] Umstattd, M. R., Motl, R., Wilcox, S., Saunders, R., & Watford, M. (2009). Measuring physical activity self-regulation strategies in older adults. *Journal of Physical Activity and Health*, *6*(s1), S105–SS112. 10.1123/jpah.6.s1.s105 19998856

[CIT0056] U.S. Department of Health and Human Services. (2018). Physical activity guidelines for Americans.

[CIT0057] Vasudevan, A., & Ford, E. (2022). Motivational factors and barriers towards initiating and maintaining strength training in women: A systematic review and meta-synthesis. *Prevention Science*, *23*(4), 674–695. 10.1007/s11121-021-01328-2 34800250 PMC9072266

[CIT0058] Wang, X., & Cheng, Z. (2020). Cross-sectional studies: Strengths, weaknesses, and recommendations. *Chest*, *158*(1), S65–S71. 10.1016/j.chest.2020.03.012 32658654

[CIT0059] Williamson, D. F., Kahn, H. S., Remington, P. L., & Anda, R. F. (1990). The 10-year incidence of overweight and major weight gain in US adults. *Archives of Internal Medicine*, *150*(3), 665–672. 10.1001/archinte.1990.00390150135026 2310286

[CIT0060] Winett, R. A., Davy, B. M., Savla, J., Marinik, E. L., Kelleher, S. A., Winett, S. G., Halliday, T. M., & Williams, D. M. (2015). Theory-based approach for maintaining resistance training in older adults with prediabetes: Adherence, barriers, self-regulation strategies, treatment fidelity, costs. *Translational Behavioral Medicine*, *5*(2), 149–159. 10.1007/s13142-015-0304-5 26029277 PMC4444707

[CIT0061] Zheng, Y., Manson, J. E., Yuan, C., Liang, M. H., Grodstein, F., Stampfer, M. J., Willett, W. C., & Hu, F. B. (2017). Associations of weight gain from early to middle adulthood with major health outcomes later in life. *The Journal of the American Medical Assoication*, *318*(3), 255–269. 10.1001/jama.2017.7092 PMC5817436 28719691

